# Templated Synthesis of SiO_2_ Nanotubes for Lithium-Ion Battery Applications: An In Situ (Scanning) Transmission Electron Microscopy Study

**DOI:** 10.1021/acsomega.2c06298

**Published:** 2022-12-28

**Authors:** Oskar Ronan, Ahin Roy, Sean Ryan, Lucia Hughes, Clive Downing, Lewys Jones, Valeria Nicolosi

**Affiliations:** † Centre for Research on Adaptive Nanostructures and Nanodevices (CRANN) and Advanced Materials and Bioengineering Research (AMBER), School of Chemistry, Trinity College Dublin, DublinDublin 2, Ireland; ‡ Advanced Microscopy Laboratory (AML), and Advanced Materials and Bioengineering Research (AMBER), Trinity College Dublin, DublinDublin 2, Ireland; § Materials Science Centre, Indian Institute of Technology, Kharagpur721302, West Bengal, India; ∥ School of Physics, Advanced Microscopy Laboratory (AML), and Advanced Materials and Bioengineering Research (AMBER), Trinity College Dublin, DublinDublin 2, Ireland

## Abstract

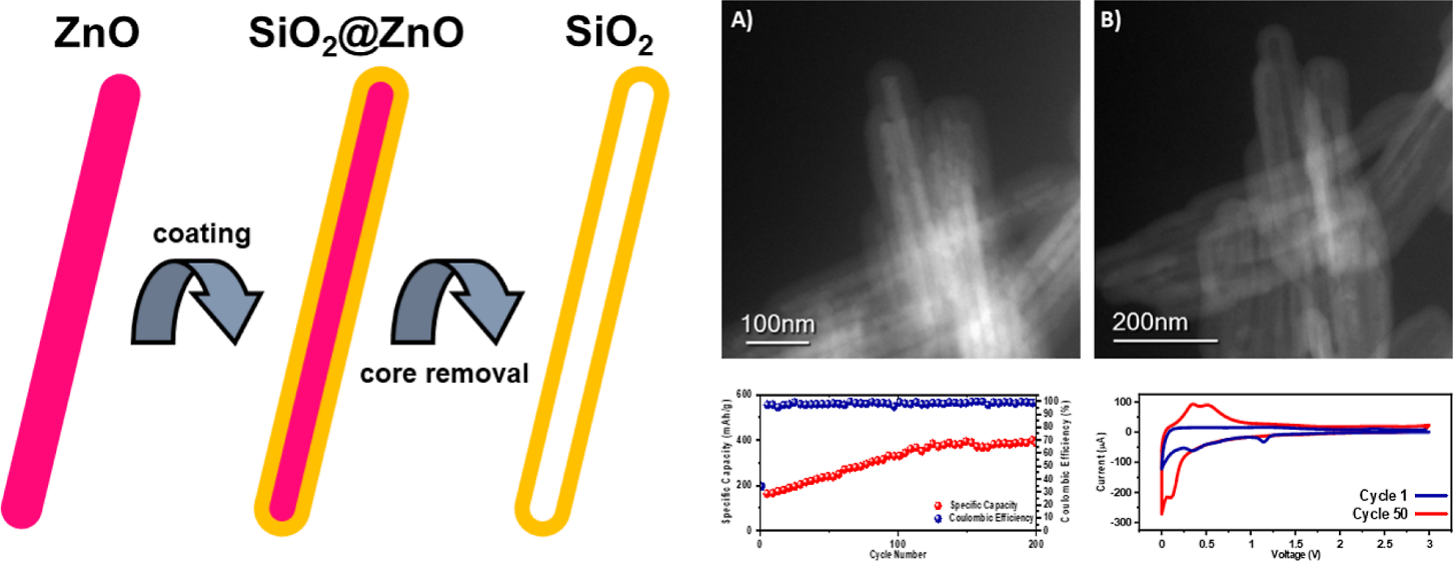

One of the weaknesses of silicon-based batteries is the rapid deterioration of the charge-storage capacity with increasing cycle numbers. Pure silicon anodes tend to suffer from poor cycling ability due to the pulverization of the crystal structure after repeated charge and discharge cycles. In this work, we present the synthesis of a hollow nanostructured SiO_2_ material for lithium-ion anode applications to counter this drawback. To improve the understanding of the synthesis route, the crucial synthesis step of removing the ZnO template core is shown using an in situ closed gas-cell sample holder for transmission electron microscopy. A direct visual observation of the removal of the ZnO template from the SiO_2_ shell is yet to be reported in the literature and is a critical step in understanding the mechanism by which these hollow nanostructures form from their core–shell precursors for future electrode material design. Using this unique technique, observation of dynamic phenomena at the individual particle scale is possible with simultaneous heating in a reactive gas environment. The electrochemical benefits of the hollow morphology are demonstrated with exceptional cycling performance, with capacity increasing with subsequent charge–discharge cycles. This demonstrates the criticality of nanostructured battery materials for the development of next-generation Li^+^-ion batteries.

## Introduction

With the increased demand for long-lasting consumer electronic devices, research on advanced energy-storage materials for lithium-ion batteries (LIBs) has exploded in recent years.^1−4^ One of the most researched and attractive potential LIB anode materials is silicon due to its almost 10-fold higher theoretical capacity (∼3579 mA h/g) over the standard graphite anode material (∼372 mA h/g).^4−9^ Pure silicon anodes tend to suffer from poor cycling ability due to the pulverization of the crystal structure after repeated charge and discharge cycles owing to the massive volumetric expansion upon lithiation (∼400%).^10^ A possible solution to this issue is the use of amorphous silicon oxide anode instead.^11−19^ Amorphous SiO_2_ has a high theoretical specific capacity of 1965 mA h/g while having no long-range crystal structure to begin with.^13,15^ With the anode remaining amorphous throughout its lifetime, Li^+^ ions are able to be inserted and removed from the anode without breaking down the material. Among various suitable morphologies of Si-based charge-storage materials, a nanostructured hollow morphology has demonstrated potential as a LIB anode material because of its high volume, low density, and ability to accommodate the large volume changes associated with the Li–Si alloying process. To achieve such a nanostructure, one such strategy is the formation of a core–shell (core-SiO_2_) structure followed by heating in a reducing atmosphere to facilitate Kirkendall diffusion to achieve hollow silicates.^20^ Mostly, physical processes such as solid-vapor process and atomic layer deposition have been reported in the literature for the core–shell nanostructure fabrication.^21,22^ The kinetic barrier for such diffusion is quite high requiring a high reaction temperature (typically >900 °C), and usually noble metal nucleation on the core is found to lower the barrier.^22,23^ Both the problems (namely, silicate formation rather than SiO_2_ and high activation energy barrier) can be circumvented in case a templated etching approach is taken into consideration, wherein the core of the SiO_2_ coated structure is etched away to give rise to hollow SiO_2_—which has higher volumetric storage capacity compared to the silicates.

The templated synthesis method using ZnO as a core and SiO_2_ as a shell described is both highly tailorable (with the tube wall thickness controllable by the reaction time of the silica-coating reaction) and highly scalable in the liquid phase.^24^ This configurability is one of the benefits of this particular synthesis method as the dimensions of the material (such as the SiO_2_ wall thickness and the ZnO nanorod size by the reaction time) can be readily and easily tuned to optimize the electrochemical performance. The results presented are comparable to some of the state-of-the-art previously reported.^25−27^

In this work, we report both the in situ observations of the synthesis of hollow SiO_2_ nanorods using ZnO templates, which are subsequently removed, and the electrochemical performance of this material. The ZnO nanorods are synthesized via a wet-chemical reaction in an alkaline environment by the following reaction that has previously been described in the literature shown in eq 1(28)
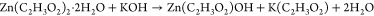

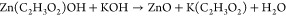
1

Following the synthesis of the ZnO nanorod templates, a SiO_2_ coating was applied via the Stöber, Fink & Bohn method.^29,30^ This sol–gel synthesis method involves the hydrolysis of the tetraethyl orthosilicate (TEOS) precursor via nucleophilic substitution of the ethoxy group (−O–C_2_H_5_) with a hydroxyl group (−OH). This is followed by the condensation reaction via nucleophilic substitution of the silanol groups (−Si–OH) to form siloxane bonds (Si–O–Si) at the surface of ZnO, which is catalyzed by the presence of ammonia (NH_3_).^30^ The overall reaction is shown below in eq 2
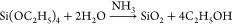
2

A direct observation of the removal of the ZnO template from the applied SiO_2_ shell is yet to be reported in the literature and is a critical step in understanding the mechanism by which these hollow nanostructures form from their core–shell precursors that we wish to probe for future electrode material design.

To study this mechanism, the main characterization technique used in this work is (scanning) transmission electron microscopy [(S)TEM]. (S)TEM was chosen due to its high spatial and real-time temporal resolution when imaging dynamically changing samples, as well as combining a variety of spectroscopic and chemical analysis techniques into a single instrument.^31−33^ For these reasons, it is an excellent technique to monitor the removal of the ZnO template from the SiO_2_ nanotubes in situ. This technique provides us with the first unique characterization insights into this synthesis mechanism by correlating a readily digestible and interpretable series of images with the spectroscopic data to observe and understand the removal of the ZnO core from the SiO_2_ shell. Placing a sample in a local gas environment inside a transmission electron microscope is not a recent technology and has been in existence since the development of one of the first environmental TEMs in the mid-1960s by Hashimoto et al.^34^ The ZnO template can be removed at elevated temperatures in a reducing atmosphere of H_2_, which can be observed in a commercial gas-cell sample holder with micro electromechanical system (MEMS) heating chips.^35−38^

Using this in situ technique, we gain a greater insight into the process that forms the nanostructures that give rise to the impressive capacity retention trend over time described in this work.

## Results and Discussion

**Figure 1:**
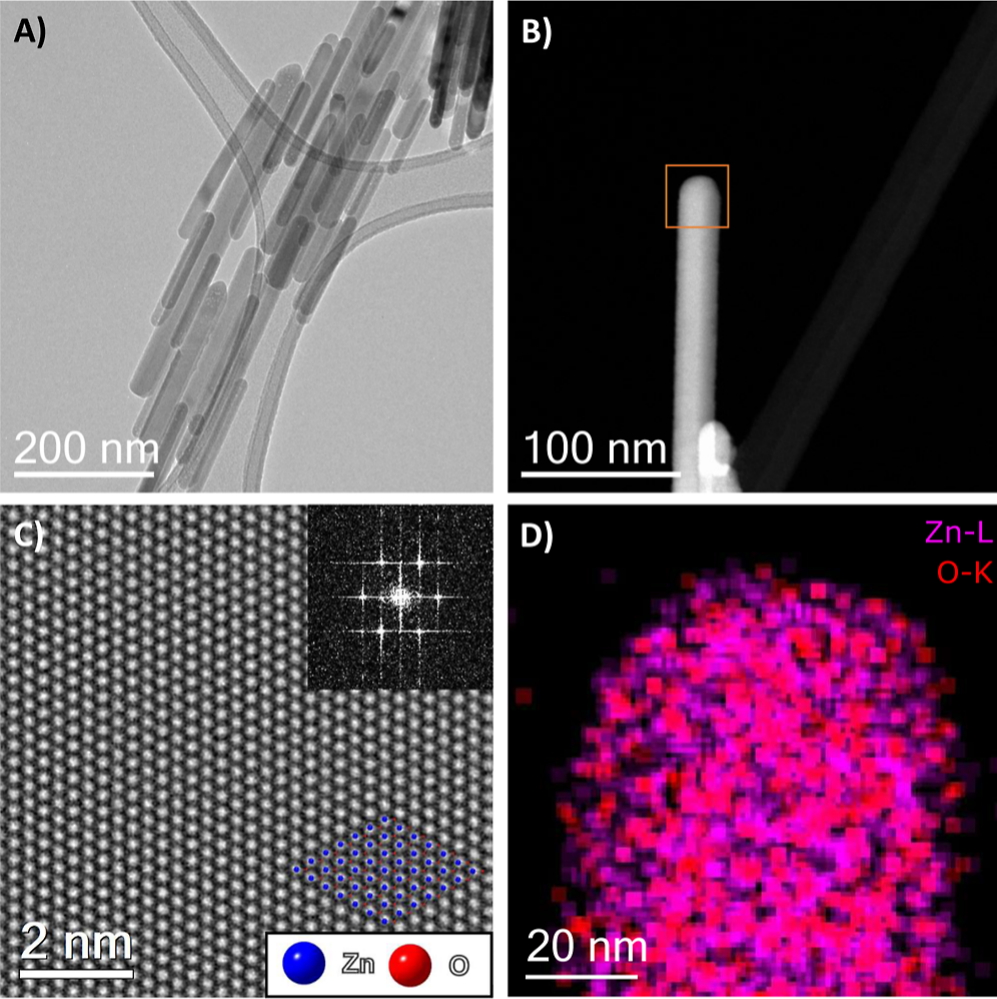
(A) Bright-field TEM of ZnO nanorods. (B) HAADF STEM image of ZnO nanorods. (C) Average background subtraction filtered rigid aligned image stack of 25 frames HAADF HR-STEM image of the ZnO nanorod structure. Wurtzite structure seen in the inset FFT. Overlaid wurtzite crystal structure of ZnO as viewed down [001] orientation (adapted from data by The Materials Project. https://materialsproject.org/).^45,46^ (D) EDXS map of highlighted ROI of ZnO nanorods in (B). Homogeneity of Zn and O within the rod visible.

The ZnO template nanostructures were first characterized by (S)TEM. As can be seen in Figure 1, the hydrothermal synthesis produces a single morphology of nanostructure, with a mean nanorod length of 173 nm (Figure S1). The size distribution shape is consistent with the nanostructure growth models for similar synthesis methods.^39−41^ High-resolution high-angle annular dark field (HAADF) STEM and the corresponding fast Fourier transform (FFT) seen in Figure 1C show the wurtzite structure of the material, and the ZnO nanorods appear monocrystalline. Energy-dispersive X-ray spectroscopy (EDX) mapping of the nanorods in Figure 1D (and the corresponding spectrum in Figure S2) confirms the chemical composition and demonstrates the homogeneity of the material.

**Figure 2:**
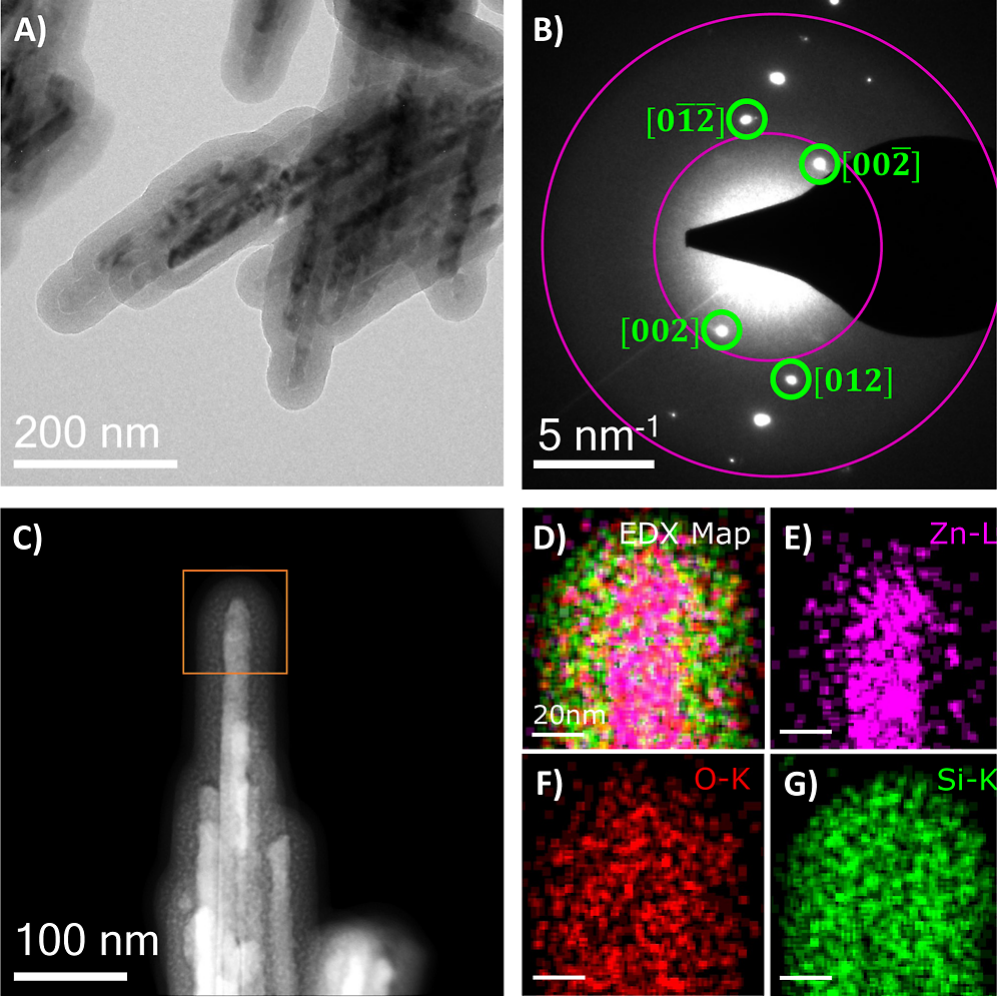
(A) Bright-field TEM image of SiO_2_@ZnO nanorods. (B) SAED showing ZnO near the [010] orientation. [002] and [012] spots and diffuse rings from the amorphous SiO_2_ layer labelled. (C) HAADF STEM of SiO_2_-coated ZnO nanorods. (D–G) EDXS maps of highlighted ROI of ZnO nanorods in (C). Core–shell structure of SiO_2_ coating of Zn and O within the rod visible from elemental distribution and image contrast.

Upon coating with SiO_2_ to an average thickness of ∼25 nm (Figure 2A,C), diffuse rings can be observed in the selected area electron diffraction (SAED) pattern of the material in Figure 2B, which indicates amorphous material alongside the crystalline ZnO (this is further confirmed by the X-ray diffraction (XRD) spectra in Figure S3, which also indicate ZnO and amorphous SiO_2_ only in the sample).^19,42−44^ EDX mapping of the SiO_2_@ZnO nanorods in Figure 2D–G (and corresponding spectrum in Figure S4) confirms the chemical composition and demonstrates the core–shell structure of the material after silica coating.

In this work, we hypothesize a mechanism for the removal of the ZnO template core based on in situ gas-cell electron microscopy. We directly observe the reduction of ZnO to Zn in a hydrogen environment via the following reaction in eq 3
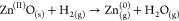
3

**Figure 3:**
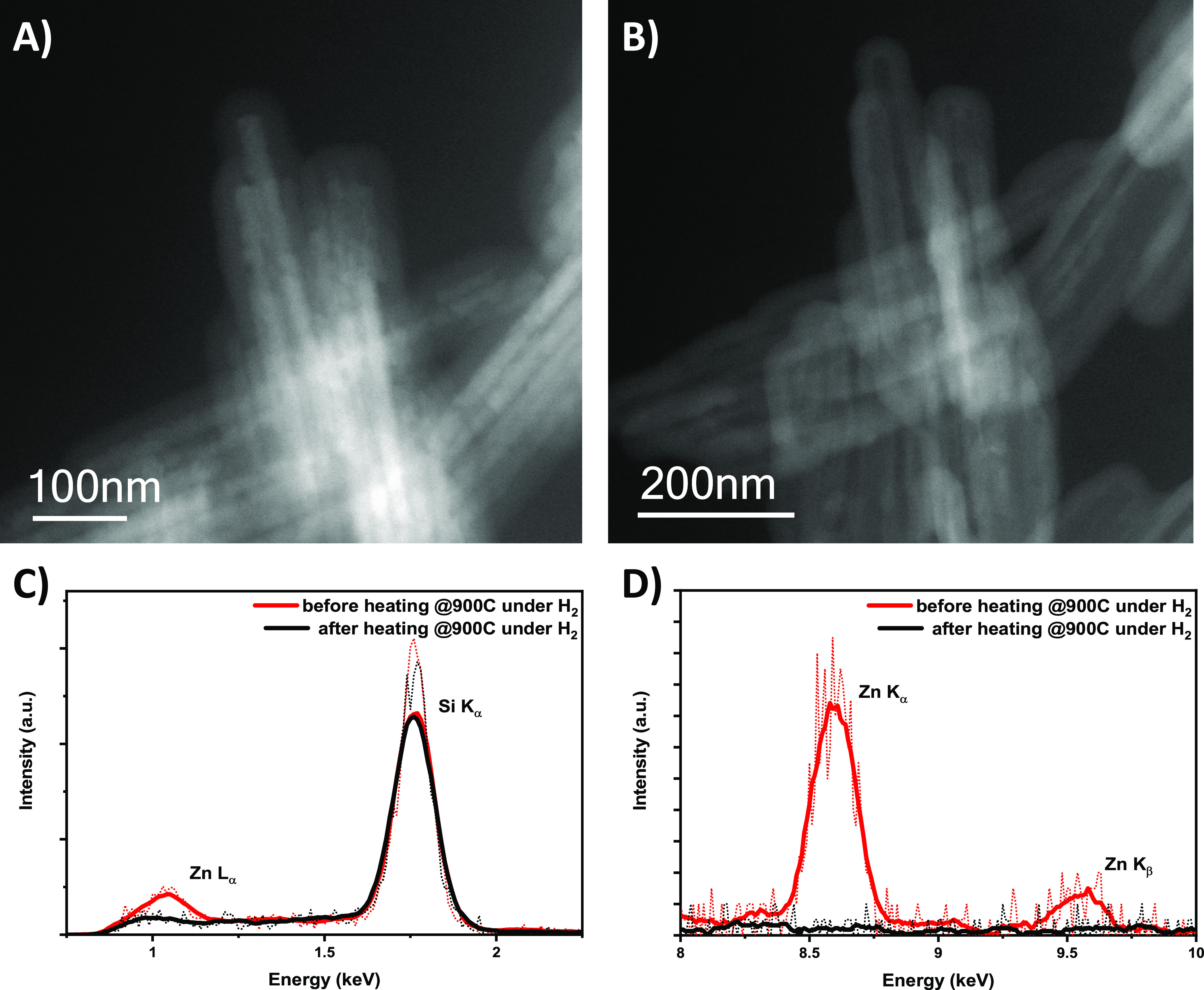
HAADF STEM of SiO_2_@ZnO nanorods (A) before in situ heating and (B) after heating at 900 °C in a 5% H_2_ atmosphere inside the nanoreactor gas-cell sample holder. The hollow structures are visible as a result of removal of the Zn core through the porous SiO_2_ coating. This is confirmed by EDX spectra in (C,D) showing the disappearance of the Zn L_α_ and K_α_ peaks at 1.012 and 8.63 keV, respectively, after heating under a reducing atmosphere.

At the experimental parameters of 900 °C and 1 bar, the amorphous porous silica layer allows the H_2_ to bind to the surface of the ZnO via heterolytic chemisorption at elevated temperatures before reacting and reducing the Zn^2+^.^47−54^ Within the scope of this experiment, a direct reduction process in which ZnO is reduced to metallic Zn vapor without an intermediate liquid phase in the presence of H_2_ in the reducing gas mixture can be observed.^55,56^ This is consistent with the literature experimental values for the vaporization point of Zn.^57,58^ The constant loss of Zn from the surface through the porous SiO_2_ shell exposes more ZnO for reduction, resulting in total removal of the ZnO core without damaging or altering the nanostructure of the SiO_2_ coating (see Figure 3 and Movie S1). The ZnO core is completely removed, while the silica remains intact as a result of this reaction, as evidenced by the disappearance of the Zn K_α_, K_β_, and L_α_ peaks at 8.63, 9.57, and 1.012 keV, respectively, and retention of the Si K_α_ peak at 1.739 keV from the simultaneously captured EDX spectra during the experiment shown in Figure 3C,D.

This reaction occurs below the bulk melting point of ZnO (1975 °C/2248.15 K) and SiO_2_ (1710 °C/1983.15 K),^59^ although literature sources demonstrate far lower melting points for nanostructured ZnO due to the nanoscale melting point depression phenomenon.^60−64^ Control experiments carried out at 900 °C under vacuum demonstrate no change in the morphology of the SiO_2_@ZnO nanorods after heating alone, indicating the key role the reducing atmosphere plays in the process (see Figure S5).

The hollow morphology of the SiO_2_ nanotubes produced by this high-temperature gas-phase process upon removal of the zinc template core has the potential to accommodate the volume changes experienced by Si-based anodes during lithiation/delithiation and promote preservation of the solid electrolyte interphase (SEI). This potential as a LIB anode was then investigated.

**Figure 4:**
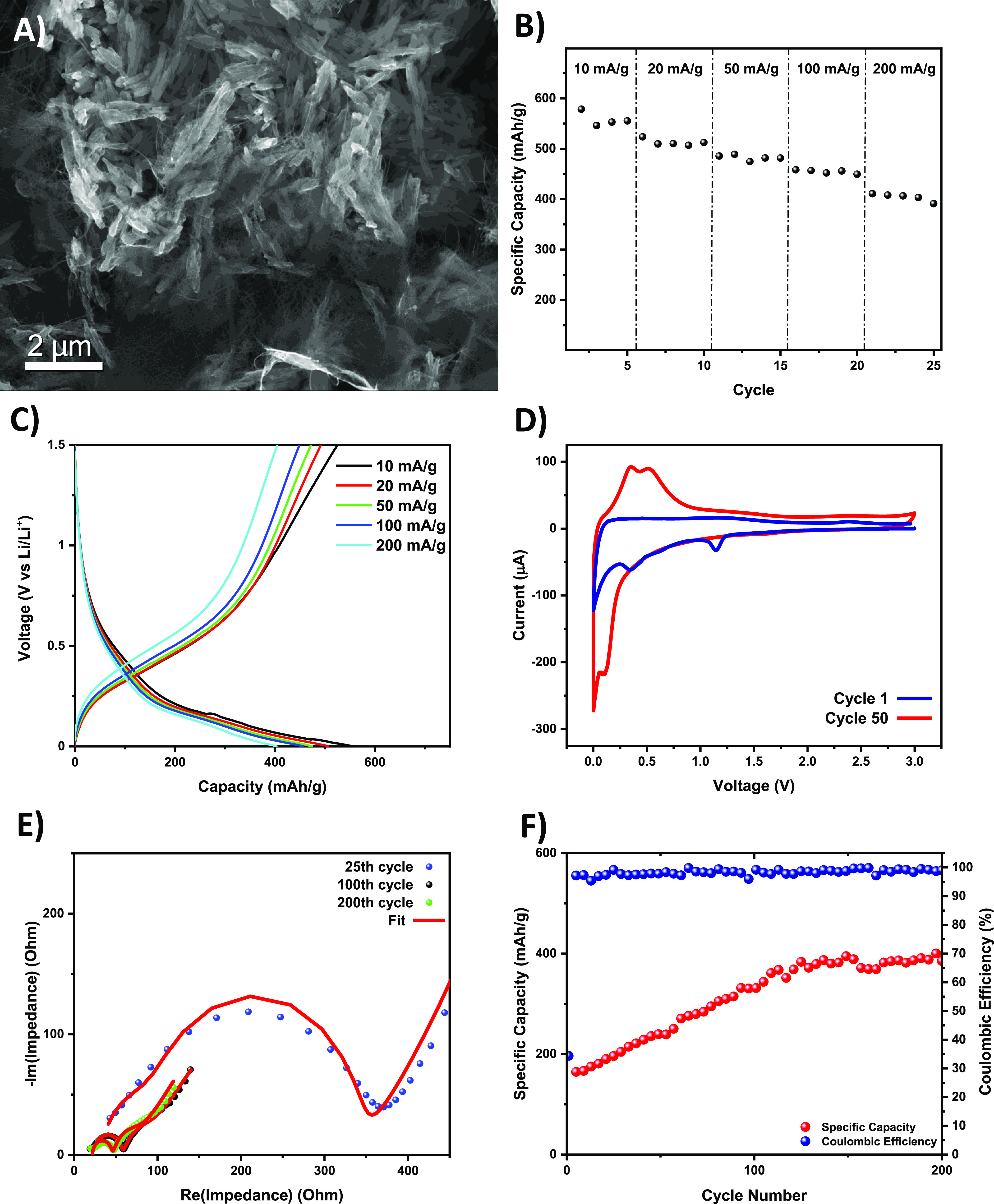
(A) InLens SEM image of the hollow SiO_2_NT/CNT electrode acquired at 10 keV. (B) Rate performance of the SiO_2_NT/CNT electrode in a Li^+^-ion battery coin cell in a range of current densities. (C) Representative galvanostatic charge–discharge voltage profiles at the fourth cycle at each current density (10–200 mA/g). (D) CV of the SiO_2_NT/CNT electrode at a cycling rate of 0.1 mV/s. (E) EIS Nyquist plot of the SiO_2_NT/CNT electrode with the equivalent circuit (inset) fitting at different cycling intervals. (F) Capacity cycling at 0.2 A/g over 200 cycles and corresponding Coulombic efficiency per cycle.

Electrochemical performance of the 25% carbon nanotube (CNT)/SiO_2_ electrode is presented in Figure 4. Rate performance, galvanostatic charge–discharge, cyclic voltammetry (CV), and electrochemical impedance spectroscopy (EIS) tests of various samples were conducted.

From the CV plot shown in Figure 4D, it was observed that the SiO_2_/CNT electrode is electrochemically active in this voltage window, with a pair of broad reversible alloying peaks revealed at an onset of ∼0.5 V versus a lithium counter electrode. The curves become highly stable immediately after the first cycle (shown in Figure S6), indicating that the high degree of reversibility of the electrochemical reactions takes place after the second cycle. The broad anodic peak is due to the alloying of amorphous Si and Li and is in agreement with the alloying–dealloying reaction between SiO_2_ and the Li–Si alloys found in the literature.^25,44^ The additional irreversible cathodic peak seen at 0.8 V in the first cycle in Figure S6 can be attributed to the formation of the SEI.^65,66^

While the SiO_2_ nanotubes are percolated with single-walled CNTs (SWCNTs) (seen in the scanning electron microscopy (SEM) image in Figure 4A), both the Li^+^-ion diffusion and electron transport kinetics within the electrode are initially sluggish, with the material performing better at lower current densities as seen in Figure 4B,C. The SiO_2_/CNT electrode exhibits an initial active mass capacity up to 580 mA h/g at 10 mA/g and decreases to 391 mA h/g at 200 mA/g after five cycles at this current density. Initial SiO_2_ electrochemical performance is thought to be limited by the internal resistance of the material and is confirmed by EIS in Figure 4E. The low initial discharge efficiency of 34.4% of the material seen in Figure S7 is suspected to be due to the initial formation of the SEI and the initially high internal resistance as evidenced by EIS in Figure 4E.^26,67,68^

The significant reduction of the total cell resistance (and conversely, increase in ionic conductivity) from the initial EIS to the EIS carried out after 100 and 200 cycles seen in Figure 4E indicates “electrochemical activation” of the electrode by cycling.^69,70^ Impedance fit calculated using equivalent circuit shown in Figure S8 shows a decrease of the total resistance () from 344.5 to 63.21 Ω (where *R*_1_ is the equivalent series resistance for the electrolyte, current collectors, and electrode materials, *R*_2_ is the interfacial resistance between the CNTs and SiO_2_ nanorods, *R*_3_ is the SEI resistance, and *R*_4_ is the charge-transfer resistance at the interface between the electrolyte and active materials).^71^ This is believed to be due to initial lithiation cycles reconstructing the electrode material to allow for easier Li^+^-ion migration within the hollow nanorods, enhancing the degree of electrochemical utilization.^27^ Loss of Li^+^-ions from the electrolyte to the anode material over cycles can form an amorphous Li_2_O–SiO_2_ glass from the reduction of SiO_2_ in the presence of Li. This is known to be an ionic-conductive solid electrolyte, thus increasing performance over time.^72−75^ This continuous increase in conductivity as the cycle number increases as evidenced by EIS (Figure 4E) corresponds to the continuous increase in capacity of the material over time. The formation of Li_2_O– SiO_2_ glass from reduction of SiO_2_ also creates local clusters of high-capacity Si for subsequent lithiation. This mechanism is described in detail by Ban et al.^73^ The higher capacity Si contributes to the increasing capacity as more SiO_2_ is reduced and the amount of Si present in the material increases.^26,73^ This is evidenced from the appearance of sharper Si lithiation peaks in the CV in later cycles, emphasizing the alloying peak at 0.15 V and the dealloying peak located at 0.34 V which are characteristic peaks of the lithiation of Si and formation of amorphous Li_*x*_Si phases.^8,9,65^ This structure decomposes upon charging back to SiO_2_, as evidenced by the high reversibility of the CV and the high Coulombic efficiency shown in Figure 4.

Hollow SiO_2_ nanotubes show exceptional cycling stability, not only retaining the capacity and nanostructure after cycling but even increasing the capacity by up to 150% after 200 cycles as seen in Figures 4F, S9, and S10. This continuous increase in performance with increasing cycle life demonstrates the benefit of the hollow morphology, namely, resistance to pulverization effects from repeated electrochemical cycling seen in more traditional bulk SiO_2_ electrodes.^19^ CV also suggests that more Li^+^ ions can be extracted from the hollow SiO_2_ nanorods in later cycles when compared to initial discharging (Figure 4D).

## Conclusions

In summary, we have studied the synthesis mechanism via gas-phase in situ (S)TEM of hollow SiO_2_ nanorods for use in energy-storage applications. The removal of the ZnO core template step of the synthesis process is observed for the first time. The criticality of a reducing atmosphere in the synthesis route was demonstrated, and mechanism is shown utilizing in situ electron microscopy in real time. The hollow SiO_2_ nanostructures demonstrate promise as an electrode material which reacts reversibly with Li^+^ ions and at low potentials versus Li^+^/Li with a wide operating voltage window. SiO_2_ shows potential as an alternative high-capacity battery anode material due to its electrochemical performance, cost, environmental impact, and stability.^19^ The low density of SiO_2_ is also promising for increasing the power density of full devices.^76^ Furthermore, the behavior of resistance to degradation and the increasing capacity of ∼150% over hundreds of cycles is highly impressive by the standard of the current state-of-the-art for this material.

## Methods

### Synthesis of ZnO Nanorods

The synthesis of the ZnO nanostructures is adapted from both Tripathi et al. and Pacholski et al.^24,77^ 14.75 g of zinc acetate dihydrate [Zn(C_2_H_3_O_2_)_2_·2H_2_O] was uniformly dispersed in 62.5 mL of methanol (23.6 mg/mL) in a round-bottomed flask by stirring for 30 min on a heating mantle maintained at 65 °C. To this, 7.4 g of KOH dissolved in 32.5 mL of methanol (22.77 mg/mL) was added slowly via a dropping funnel under vigorous stirring (Figure S11A). The mixture was heated until the volume had reduced by 60%. The solution was then transferred to a PTFE vessel, sealed in a stainless-steel hydrothermal autoclave (Parr Instrument Company, USA. model 4744) and heated at 120 °C for 6 h. The white precipitate obtained after cooling was washed eight times first with methanol and then with DI water and dried under vacuum at 80 °C for a minimum of 2 h.

### Silica Coating of ZnO Nanorods

The silica coating over the ZnO nanorods was performed by the Stöber process detailed previously.^30^ ZnO nanorods (0.2 g) were dispersed in a mixture of 9 mL of deionized water and 20 mL of ethanol by ultrasonication for 30 min. Once dispersed, 0.5 mL of 25% ammonia solution and 0.5 mL of TEOS (TEOS/Si(C_2_H_5_O)_4_) were added, and the solution was stirred for 3 h (Figure S11B). (∼6.7 mg/mL of ZnO to total volume; 40:18:1:1 H_2_O/CH_3_OH/NH_3_/TEOS v/v %) The final product was collected by centrifugation and washed with DI water and ethanol several times. The sample was dried under vacuum at 80 °C for a minimum of 2 h.

### Synthesis of Hollow SiO_2_ Nanorods

SiO_2_-coated ZnO (SiO_2_@ZnO) nanorods were baked in a tube furnace at 900 °C under 95:5 vol % N_2_/H_2_ atmosphere at 1 bar for 1 h to allow for the complete removal of ZnO.

### Electron Microscopy Characterization

The ZnO, SiO_2_@ZnO, and SiO_2_ nanorods were observed via field-emission (S)TEM (Titan, Thermo Fisher Scientific Inc.). The macrostructure and elemental composition of the ZnO and SiO_2_@ZnO nanorods were analyzed using (S)TEM coupled with simultaneous EDX (Bruker QUANTAX XFlash 6T-30 30 mm^2^ EDXS detector), and an acceleration voltage of 300 kV was employed for both TEM and STEM imaging during the measurements. STEM images were acquired using a beam current of 0.5 nA and a probe dwell time of 20 μs/px.

TEM images were processed using DigitalMicrograph (DM) (Gatan Inc., USA), and rigid registration image alignment of fast-acquisition multiframe STEM images to increase the signal/noise ratio was performed using the SmartAlign plugin over 25 frames.^78^

The samples were prepared by dispersing the synthesized materials in DI water via ultrasonic bath sonication (Fisherbrand 11207 operated at 37 kHz).^79^ The dispersion was deposited on a lacey carbon 400 mesh Cu grid (01896-F, TED PELLA Inc.) by dropcast for observation and allowed to dry in vacuum.

Bulk electrodes were observed via SEM (Zeiss GEMINI 0.1–30 kV, Carl Zeiss Microscopy, LLC, USA).

### In Situ Observations

SiO_2_@ZnO nanorods were deposited onto Climate E-chips (P.T.GH.SS.2, DENS Solutions B.V. Delft, Netherlands) shown in Figure S12 in the same manner. Each chip, which consisted of an ∼30 nm-thick SiN_*x*_ membrane, was mounted in a Climate TEM holder (DENS Solutions B.V. Delft, Netherlands). Here, the SiN_*x*_ membrane acts as the sample support while simultaneously isolating the sample from the vacuum environment and allowing for the introduction of the H_2_ atmosphere and a precise feedback-loop-based temperature control from the MEMS heating coil inside the nanoreactor.^35,37^

The sample was loaded into the microscope, and a nanorod was chosen as the region of interest (ROI) for imaging. The sample was heated to 900 °C under 95:5 vol % N_2_/H_2_ atmosphere at 1 bar until the ZnO cores had been removed.

In situ image series were acquired in STEM using a beam current of 0.5 nA and a probe dwell time of 4 μs/px.

In situ STEM image stacks were processed using DM, and postacquisition drift correction of the image stack was processed using SmartAlign plugin over 1182 frames.^78^

### Electrochemical Characterization

Synthesized SiO_2_ nanorods (100 mg) were combined with 0.4% Tuball-SWCNT(PVDF/NMP) (OCSiAl, S.A.) in a ratio of 3:1 w/w % and cast on a battery grade copper foil (areal loading of 1 mg cm^–2^) and allowed to dry before being placed in an oven at 40 °C for 24 h. 12 mm diameter electrodes were punched and heated under vacuum in a tube furnace with a ramp of 10 °C/min and a hold at 700 °C for 2 h to remove the NMP solvent and carbonize the PVDF binder from the as-received CNT dispersion.^80−82^ This method is described in previous works of the authors.^83^ The electrodes were assembled into a 2032 coin-type half-cell with a polyolefin separator and lithium foil acting as the counter and reference electrodes. The standard LP30 (1 M LiPF_6_ EC/DMC, 1:1) electrolyte was used. The cells were allowed to cure for 24 h at 40 °C prior to testing. Electrochemical testing of coin cells was carried out with a BioLogic BCS-805 potentiostat (BioLogic Science Instruments, France). CV was carried out using a voltage window of 0–3 V to ensure completion of all electrochemical processes. Galvanostatic charge–discharge profiles were run at 0–1.5 V after determining the active region from the CV, and a representative curve for each current density was chosen.

### XRD Characterization

Synthesized ZnO nanorods, SiO_2_@ZnO nanorods, and hollow SiO_2_ nanorods were analyzed using a Bruker D8 Discover diffractometer with a Cu Kα radiation source, a Goeble mirror, and a Ge double bounce monochromator.

## Supporting Information Available

Additional experimental details, figures, materials, and methods (PDF)
STEM movie of ZnO core removal from the SiO_2_ shell (MP4)
