# Supplementary material for: Templated Synthesis of SiO2 Nanotubes for Lithium-Ion Battery Applications: An In Situ (Scanning) Transmission Electron Microscopy Study
Source: ACS Omega. 2022 Dec 28;8(1):925–33. doi: 10.1021/acsomega.2c06298 (PMC9835544; doi:10.1021/acsomega.2c06298)
Supplement: Supplementary file 1 — ao2c06298_si_001.pdf [file ao2c06298_si_001.pdf]

Templated synthesis of SiO_2 nanotubes for lithium-ion battery applications; an *in-situ* (Scanning) Transmission Electron Microscopy study - Supplementary Information

Oskar Ronan[1,2,], Ahin Roy[2,3,*], Sean Ryan[1], Lucia Hughes[1,2], Clive Downing[2], Lewys Jones[4], and Valeria Nicolosi[1,*]*

*Corresponding authors

[1]Centre for Research on Adaptive Nanostructures and Nanodevices (CRANN) and Advanced Materials and Bioengineering Research (AMBER), School of Chemistry, Trinity College Dublin, Dublin 2, Ireland

[2]Advanced Microscopy Laboratory (AML), and Advanced Materials and Bioengineering Research (AMBER), Trinity College Dublin, Dublin 2, Ireland

[3]Materials Science Centre, Indian Institute of Technology, Kharagpur, West Bengal, India

[4]School of Physics, Advanced Microscopy Laboratory (AML), and Advanced Materials and Bioengineering Research (AMBER), Trinity College Dublin, Dublin 2, Ireland

SUPPLEMENTARY FIGURES

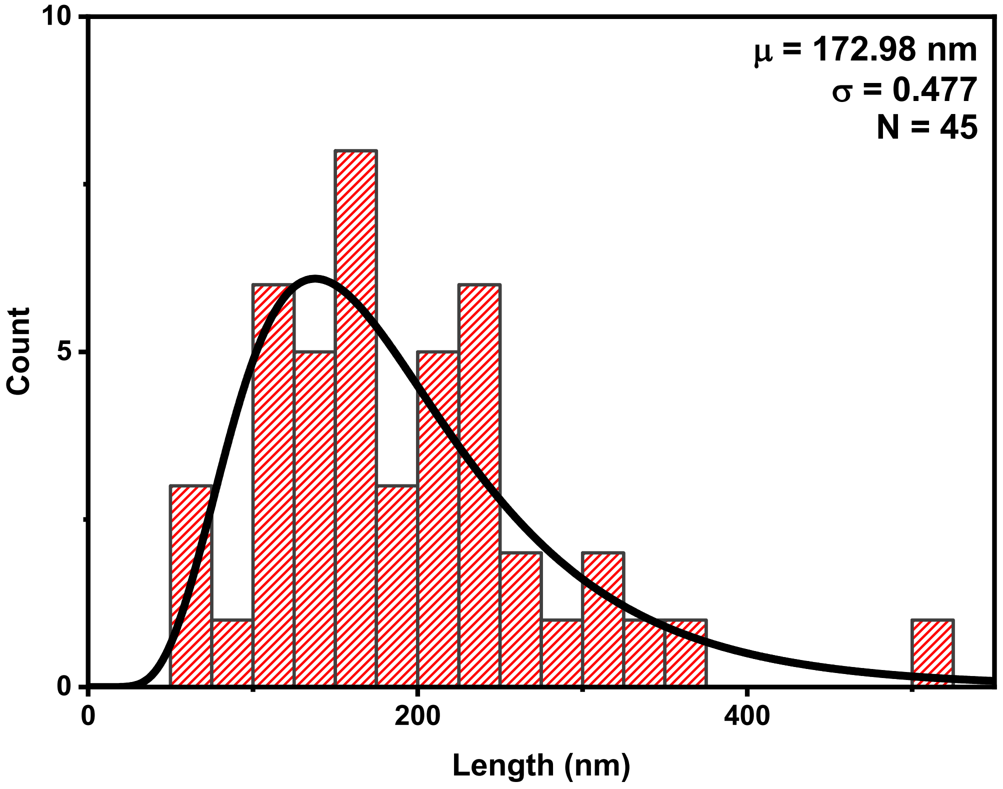

Figure S1. Lognormal size distribution analysis of synthesised ZnO nanorods. Mean rod length measured at ~173 nm. Distribution shape consistent with nanostructure growth models for this synthesis method.[39–41]

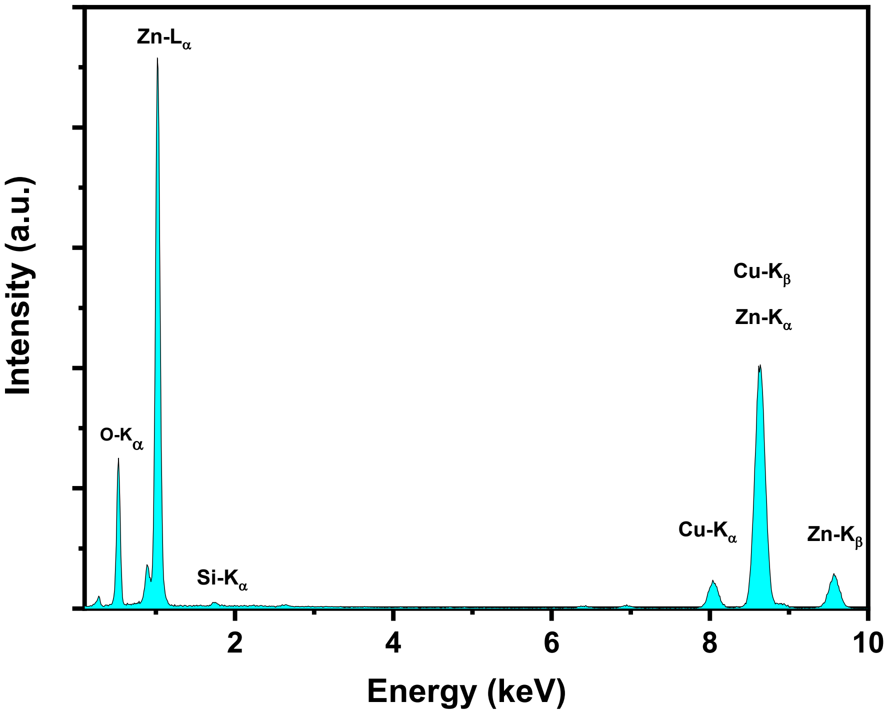

Figure S2. Integrated EDX spectrum of mapping data in Figure 1. Zn and O peaks clearly visible. (Si peak due to an EDX spectral artefact; an internal fluorescence peak from the silicon window on the silicon drift detector likely. Cu peak is a background signal from the TEM grid.)

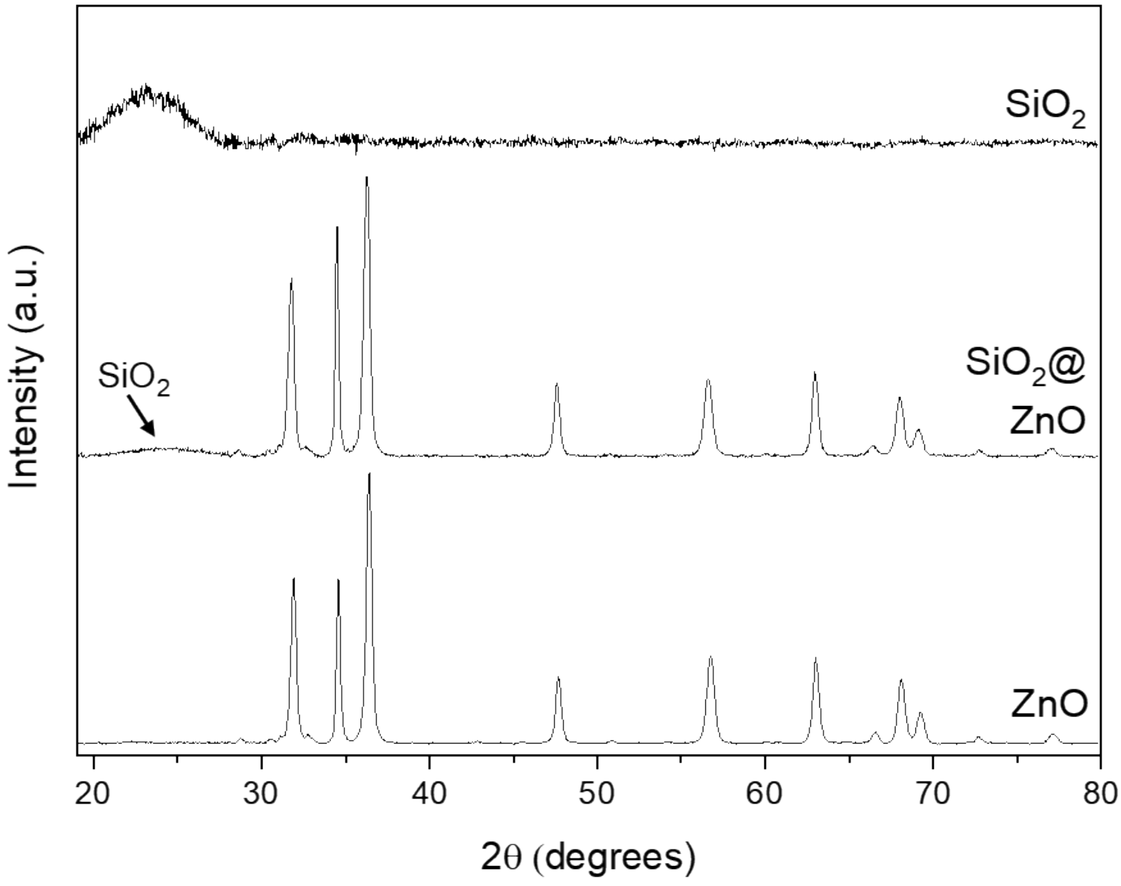

Figure S3. XRD Spectrum confirming amorphous nature of SiO_2 post total removal of the ZnO core by heating in a reducing atmosphere.

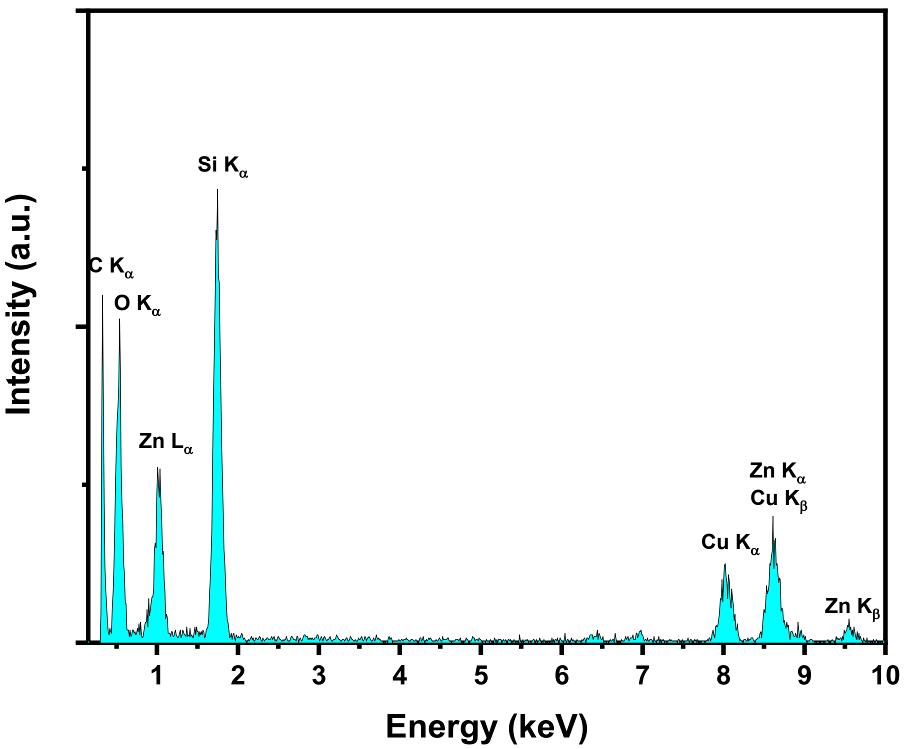

Figure S4. Integrated EDX spectrum of mapping data in Figure 2. Zn, Si, and O peaks clearly visible. (Cu peak is a background signal from the TEM grid.)

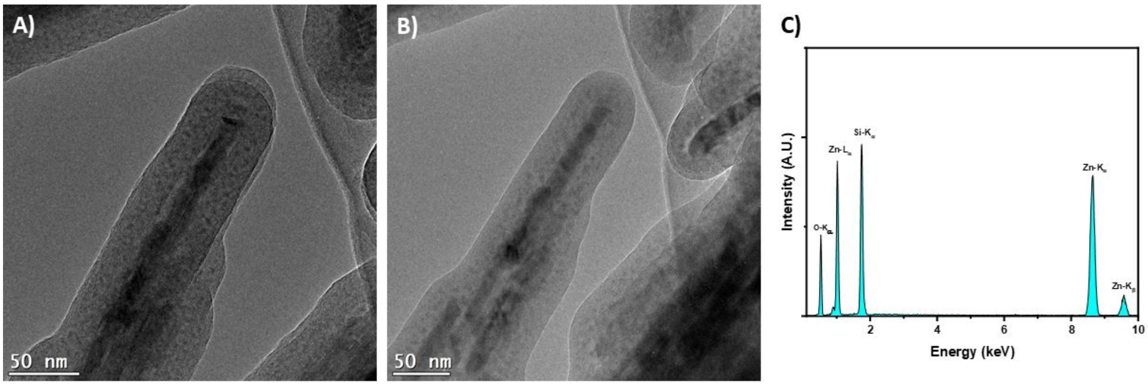

Figure S5. BF TEM images of SiO_2@ZnO nanorods A) before and B) after heating at 900˚C under vacuum on DENS Solutions Wildfire MEMS SiN_x heating chip. C) Integrated EDX spectrum of SiO_2@ZnO nanorods after control experiment. Zn peaks at 1.012keV and 8.63keV remain after heating in vacuum, indicating that H_2 is a necessary component of the ZnO removal reaction.

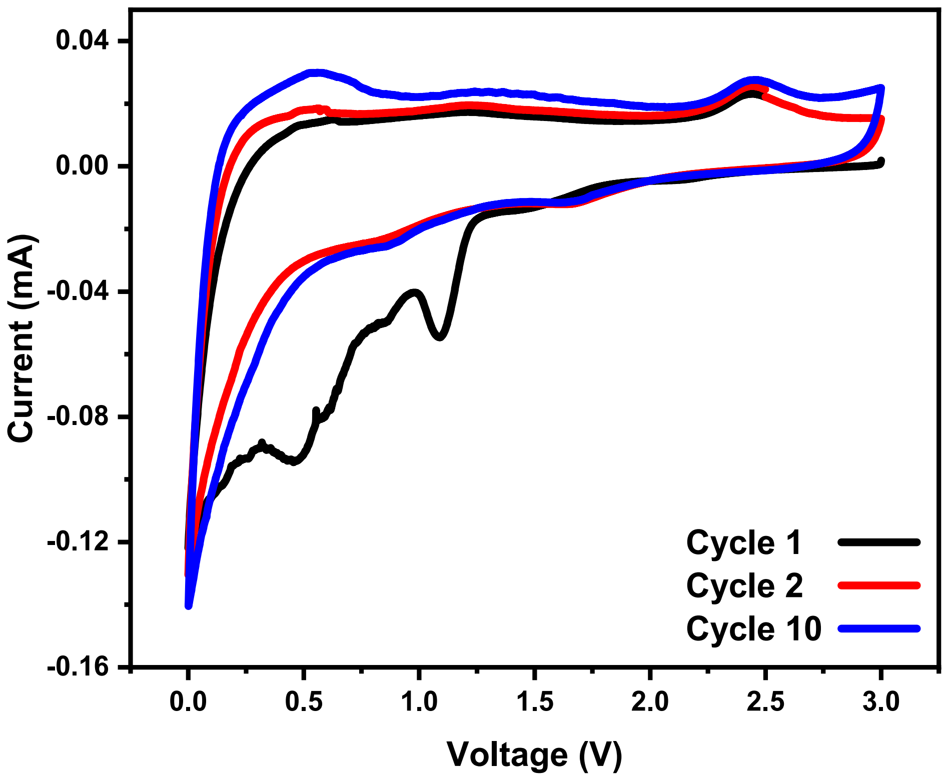

Figure S6. CV of first 10 cycles at 0.1mV/s. Irreversible cathodic peaks at 0.8V visible on first cycle only due to formation of the SEI.[65]

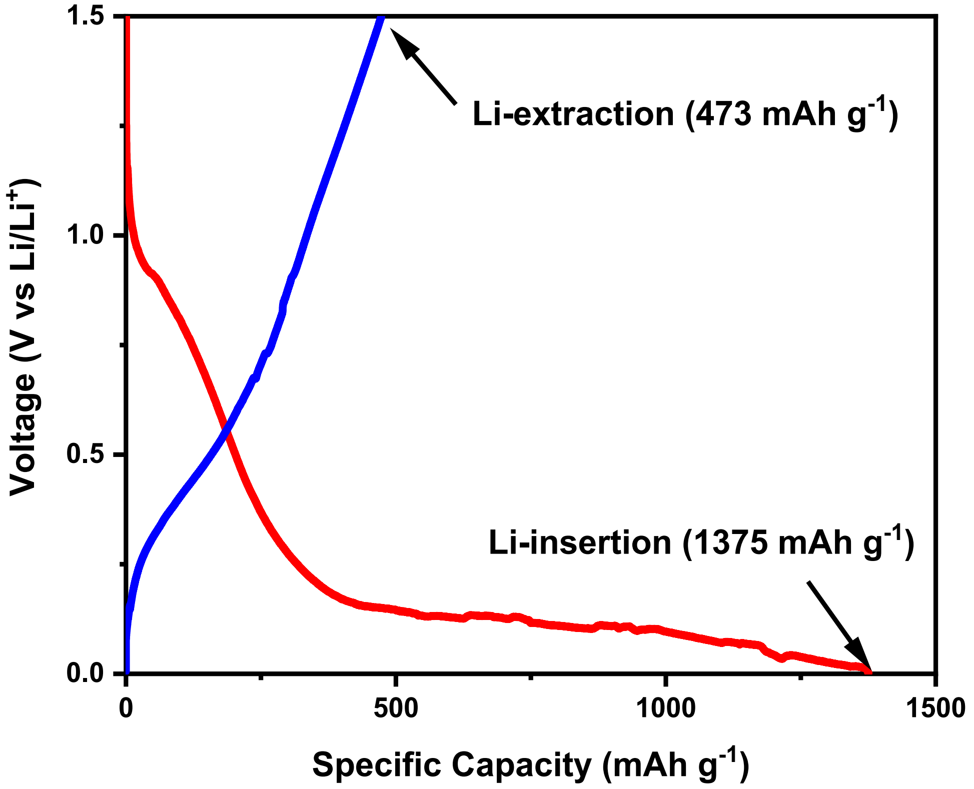

Figure S7. Initial charge-discharge curve of SiO2NT/CNT electrode. High initial charge capacity of 1375 mAh/g observed. This is approximately 70% of the theoretical capacity of the material.[15] Low discharge efficiency of 34.4% of material suspected to be due to initial formation of the SEI and Li_2O, and high internal resistance as evidenced by the initial EIS in Figure 4E.

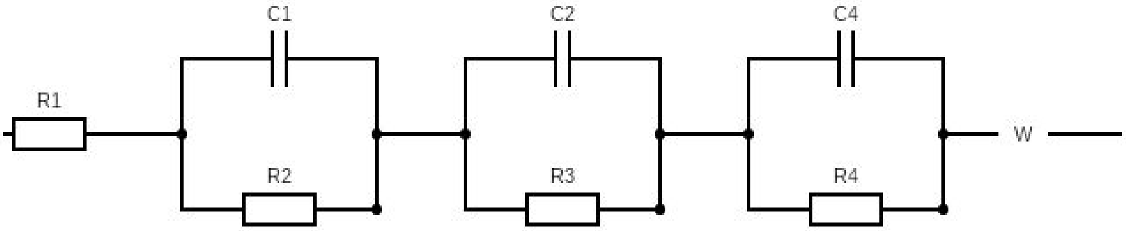

Figure S8. Equivalent circuit diagram used to calculate impedance fit for EIS in Figure 4E.

R = resistors, C = capacitors, W = Warburg Impedance.

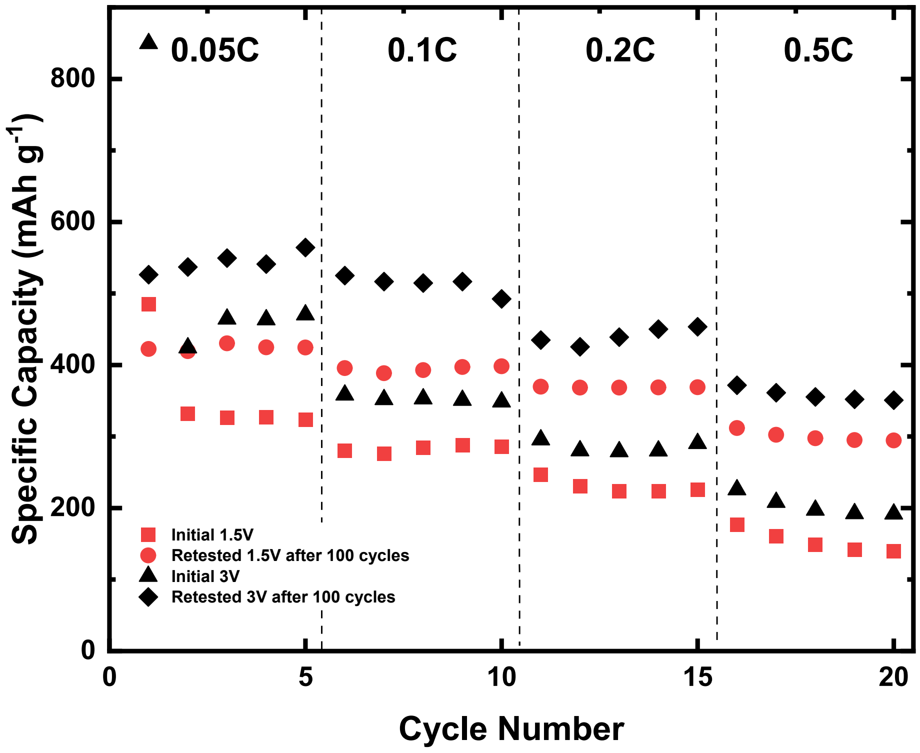

Figure S9. Rate performance testing demonstrating increase in specific capacity of SiO2 nanorods after 100 cycles at both 0-3V and 0-1.5V voltage windows.

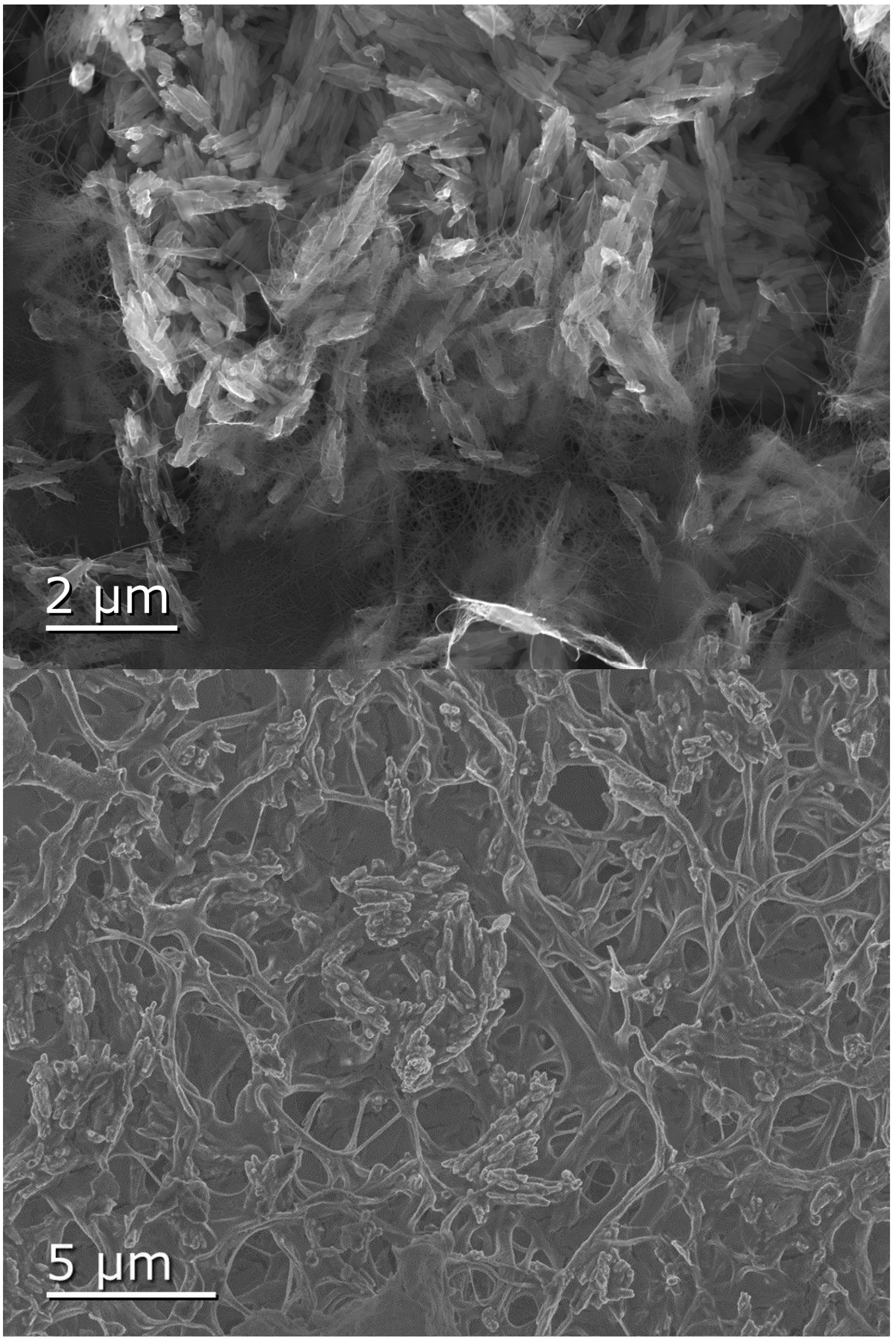

Figure S10. InLens SEM image of hollow SiO_2NT/CNT electrode acquired at 2keV before cycling and recovered from coin cell after 200 charge-discharge cycles. Nanostructured SiO_2 rods still discernable in electrode, further confirming robust electrochemical cycling performance of this nanostructure.

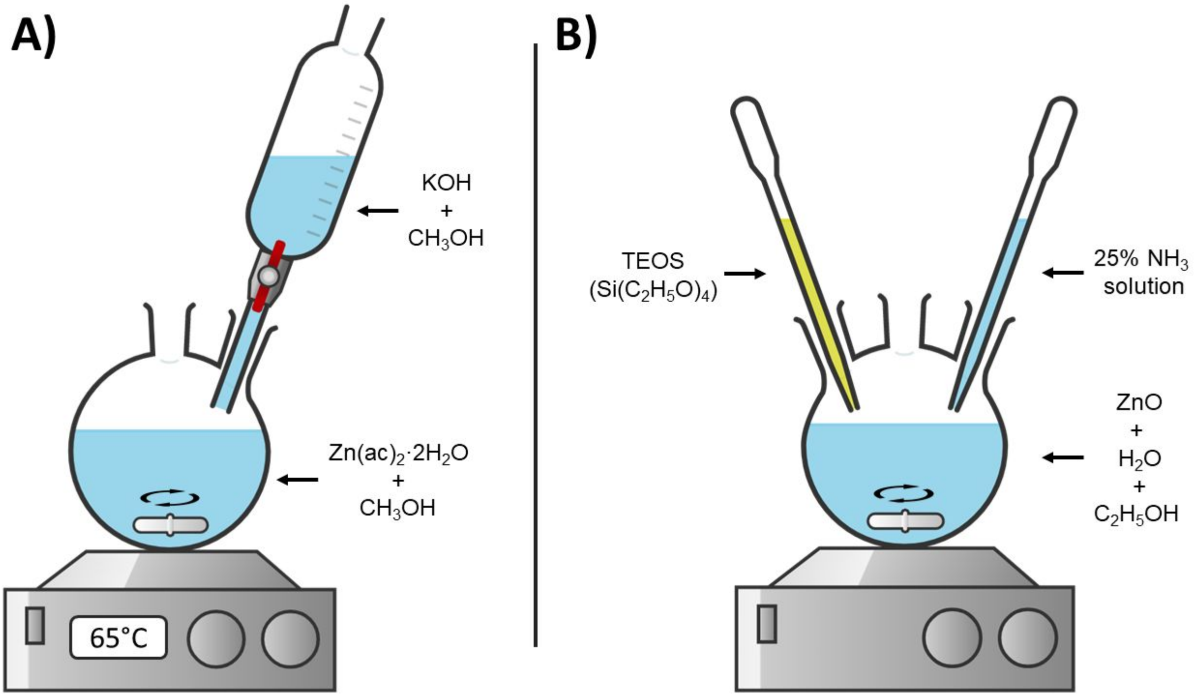

Figure S11. Schematic procedure diagram of A) ZnO synthesis and B) silica coating via Stöber process. Diagrams made in Chemix (https://chemix.org).

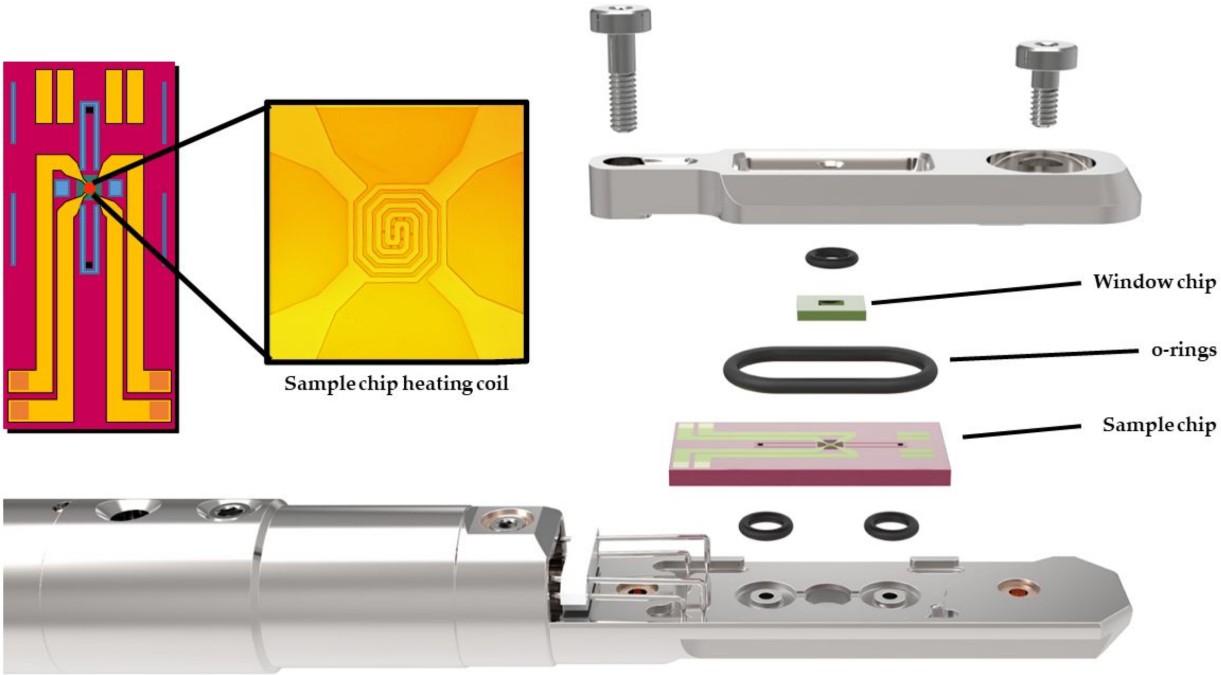

Figure S12. Exploded view schematic of DENS Solutions Climate holder and corresponding MEMS SiNx window chips. Heating coil located centrally in the window is shown. SiNx windows isolate the gas flow inside the holder from the vacuum of the TEM column and provide sample heating. (DENS Solutions B.V. Delft, Netherlands.)

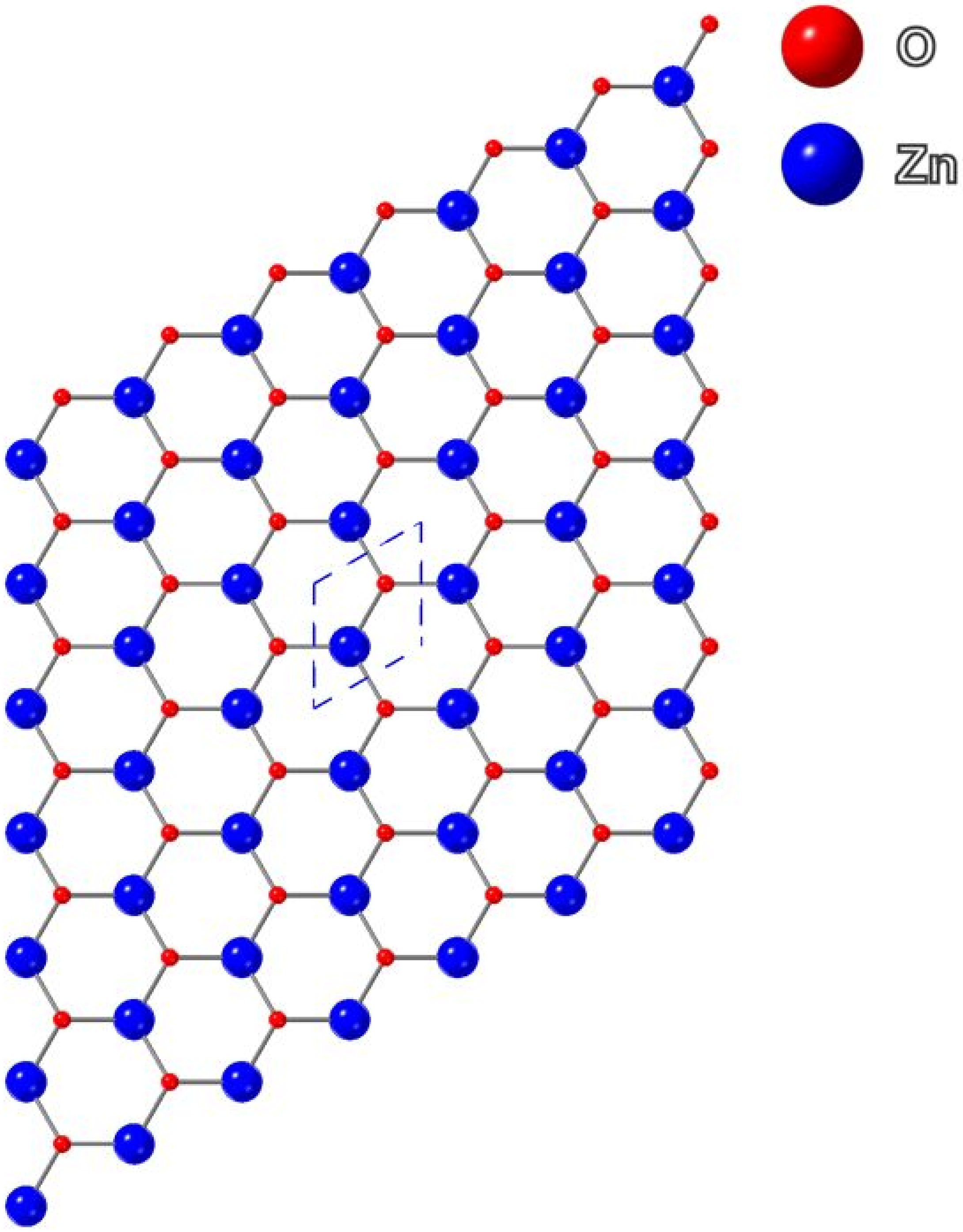

Figure S13. Hexagonal ZnO crystal structure. $P6_3mc$ space group oriented along [001]. a,b: 3.3510Å c: 5.2260Å. α,β: 90° γ: 120°. [45] Unit cell highlighted in dotted line.

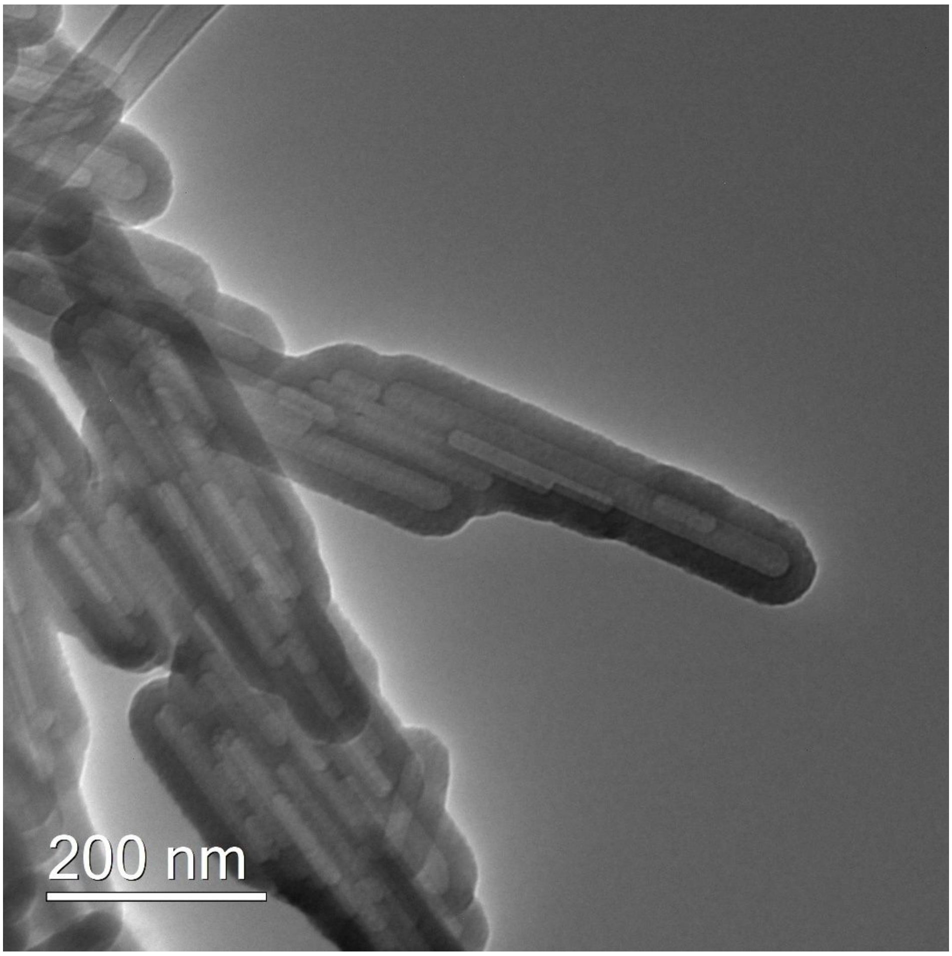

Figure S14. BF TEM images of hollow SiO_2 nanorods formed by ex-situ heating of SiO_2@ZnO nanorods under experimental conditions. This control demonstrates the reaction proceeds under reducing gas conditions in the absence of the electron beam of the *in-situ* experiment.

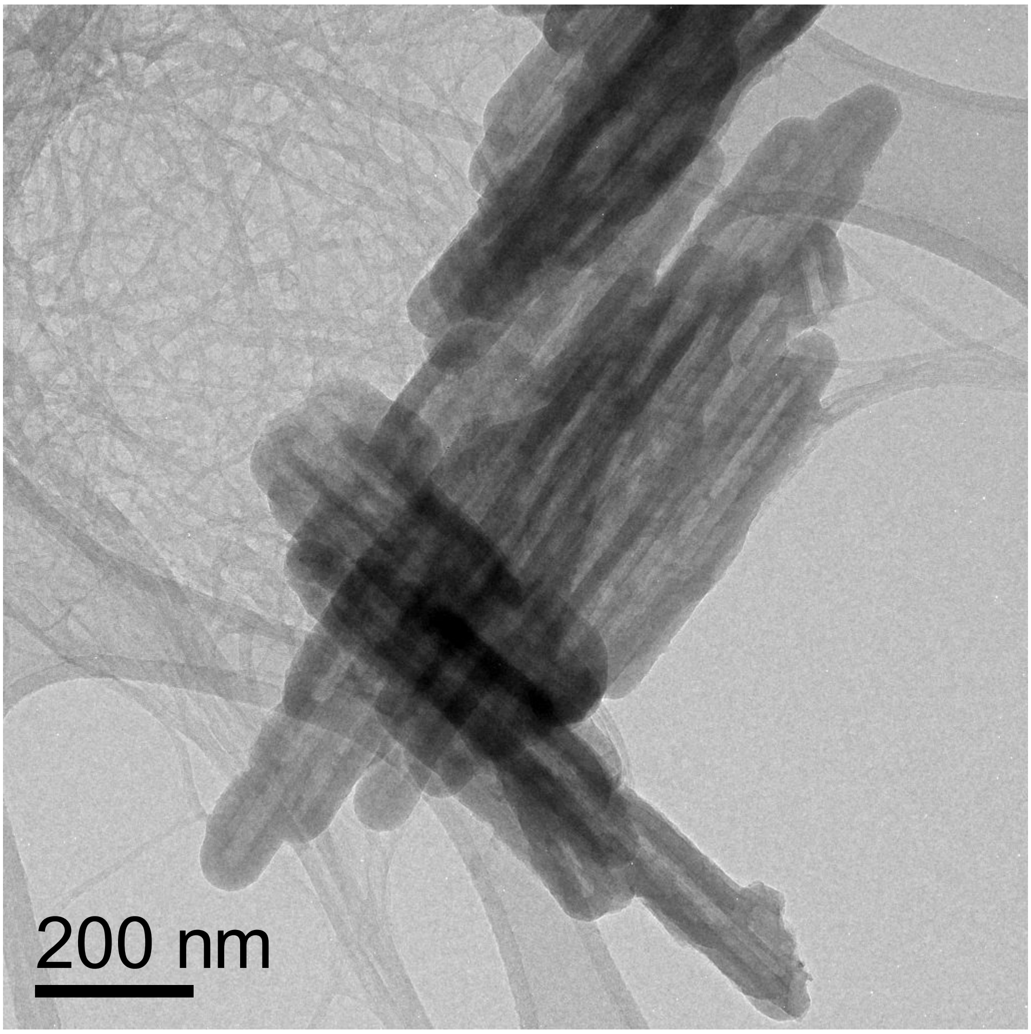

Figure S15. BF TEM image of hollow SiO_2 nanorods – CNT composite after electrochemical cycling. Morphology retained after repeated cycles.
